# Bidomain Predictions of Virtual Electrode-Induced Make and Break Excitations around Blood Vessels

**DOI:** 10.3389/fbioe.2017.00018

**Published:** 2017-03-27

**Authors:** Adam J. Connolly, Edward Vigmond, Martin J. Bishop

**Affiliations:** ^1^Department of Biomedical Engineering and Imaging Sciences, King’s College London, London, UK; ^2^IHU Liryc, Electrophysiology and Heart Modeling Instituté, Fondation Bordeaux Université, Bordeaux, France; ^3^IMB, UMR 5251, Univ. Bordeaux, Talence, France

**Keywords:** bidomain, defibrillation, cardiac electrophysiology, modeling, strength–interval, vessels, shock, low energy

## Abstract

**Introduction and background:**

Virtual electrodes formed by field stimulation during defibrillation of cardiac tissue play an important role in eliciting activations. It has been suggested that the coronary vasculature is an important source of virtual electrodes, especially during low-energy defibrillation. This work aims to further the understanding of how virtual electrodes from the coronary vasculature influence defibrillation outcomes.

**Methods:**

Using the bidomain model, we investigated how field stimulation elicited activations from virtual electrodes around idealized intramural blood vessels. Strength–interval curves, which quantify the stimulus strength required to elicit wavefront propagation from the vessels at different states of tissue refractoriness, were computed for each idealized geometry.

**Results:**

Make excitations occurred at late diastolic intervals, originating from regions of depolarization around the vessel. Break excitations occurred at early diastolic intervals, whereby the vessels were able to excite surrounding refractory tissue due to the local restoration of excitability by virtual electrode-induced hyperpolarizations. Overall, strength–interval curves had similar morphologies and underlying excitation mechanisms compared with previous experimental and numerical unipolar stimulation studies of cardiac tissue. Including the presence of the vessel wall increased the field strength required for make excitations but decreased the field strength required for break excitations, and the field strength at which break excitations occurred was generally greater than 5 V/cm. Finally, in a more realistic ventricular slice geometry, the proximity of virtual electrodes around subepicardial vessels was seen to cause break excitations in the form of propagating unstable wavelets to the subepicardial layer.

**Conclusion:**

Representing the blood vessel wall microstructure in computational bidomain models of defibrillation is recommended as it significantly alters the electrophysiological response of the vessel to field stimulation. Although vessels may facilitate excitation of relatively refractory tissue via break excitations, the field strength required for this is generally greater than those used in the literature on low-energy defibrillation. However, the high-intensity shocks used in standard defibrillation may elicit break excitation propagation from the coronary vasculature.

## 1 Introduction

Defibrillation via an implanted cardioverter defibrillator (ICD) remains the only reliable means of successfully terminating otherwise lethal cardiac arrhythmias. Despite its efficacy, the strong shocks required to ensure cardioversion render it a sub-optimal therapy, leading to the active pursuit of its refinement or novel lower energy protocols and electrode configurations. Both conventional (strong) and recently suggested low-energy defibrillation (Fenton et al., 2009; Janardhan et al., 2012; Luther et al., 2012; Rantner et al., 2013b) are thought to be driven by virtual electrodes (VEs), formed within tissue distant from the physical electrodes. VEs are produced upon application of an electric field at regions of conductivity heterogeneity in the intra-/extracellular spaces of the myocardial tissue. Electrical current then moves between the two cellular domains to redistribute itself in accordance with these changes, resulting in localized depolarization and hyperpolarization. Localized regions of depolarization within the myocardium may create new excitation wavefronts, which may act to annihilate fibrillation wavefronts and terminate the arrhythmia (Zipes et al., 1975). However, the induced regions of hyperpolarization have the ability to cause refractory myocardium to become re-excitable. These two competing effects, combined with the spatially inhomogeneous and often temporally aperiodic excitability and complex structural anatomy of the fibrillating heart, serve to complicate defibrillation strategies. Refinement of conventional ICD shocks and the advancement of novel low-energy protocols into clinical practice therefore necessitate a greater understanding of the specific mechanisms behind VE formation, particularly around fine-scale intramural anatomical structures.

Anatomically detailed modeling studies based on high-resolution MR imaging data have suggested the importance of including the coronary vasculature within computational models for simulation of strong defibrillation shocks (Bishop et al., 2010a, 2012). Such studies explicitly showed the formation of VEs around vessel cavities and highlighted important differences between including and excluding these structures on defibrillation outcomes (Bishop et al., 2012). A series of recent experimental work has also demonstrated the promising success of low-energy defibrillation protocols consisting of a series of low intensity monophasic pulses which terminated fibrillatory activity in canine preparations (Fenton et al., 2009; Luther et al., 2012). Corresponding theoretical analysis suggested that the mechanism of low-energy defibrillation was mainly driven by depolarized VEs formed around intramural blood vessels, which led to the progressive excitation of the surrounding excitable tissue, terminating the arrhythmia.

An issue, yet to be fully investigated, is the mechanism by which vessels may help activate intramural cardiac tissue which is not fully excitable (or in the refractory phase). This is particularly pertinent, as during fibrillation, wavefronts are constantly interacting with wave tails, such that very little completely recovered tissue exists, with the majority of the tissue being in a refractory or relatively refractory state. Understanding the nature of post-shock propagation under these conditions is essential in elucidating the underlying physiological processes driving low-energy defibrillation.

In the study, we first seek to quantify the field strengths at which vessels mediate secondary sources of excitation in different states of refractoriness (via “make” or “break” excitations). We compare how these field strengths relate to quoted low-energy fields as well as investigate how they depend on the specific micro-anatomy of the vessel in terms of size and structure, including the presence/absence of an insulating vessel wall. We then highlight the mechanisms by which the induced VE patterns excite refractory tissue and how this is governed by the anatomical distributions of vessels in terms of their proximity to other vessels and within the myocardial wall. Finally, we discuss the implications of the results in the context of standard and low-energy defibrillation strategies.

## 2 Materials and Methods

### 2.1 Theoretical Background

#### 2.1.1 Excitation of Relatively Refractory Tissue

The mechanisms by which relatively refractory cardiac tissue may be re-excited by a premature unipolar stimulus has been investigated in detail both in computational bidomain studies (Bray and Roth, 1997; Kandel and Roth, 2013, 2014) and corresponding optical mapping experimental work (Sidorov et al., 2005). These studies have demonstrated that the application of a unipolar stimulus to cardiac tissue induces a characteristic dog-bone VE pattern, consisting of neighboring de/hyperpolarization. This specific VE pattern forms because, in general, myocardial tissue is anisotropic to different degrees within the intra- and extracellular spaces. The different VE patterns (Dekker, 1970) from unipolar stimuli of different polarities (anodal or cathodal) cause different excitation dynamics in cardiac tissue—specifically “anode/cathode make” excitation (when applied to diastolic tissue) and “anode/cathode break” excitation (when applied to relatively refractory tissue). Break excitations occur because, if a unipolar stimulus is applied to relatively refractory tissue, the hyperpolarizing action of the stimulus acts to restore excitability to these specific regions under the virtual cathodes. At shock cessation, the depolarized tissue (under the virtual anodes) can then propagate into these post-shock excitable channels (previously hyperpolarized by the shock), initiating a “break” excitation. This initial propagation may then give time for the surrounding bulk of the tissue (previously refractory) to naturally regain excitability, facilitating propagation away from the stimulus site. Consequently, the specific VE pattern induced by a unipolar stimulus, and particularly the hyperpolarizing action, facilitates propagation into otherwise refractory tissue.

#### 2.1.2 Strength–Interval Curves

The relationship between the prematurity of the unipolar *S*_2_ pulse (corresponding to the state of tissue refractoriness) and the strength of the stimulus required to elicit propagation is quantified in the strength–interval (SI) curve. Computational bidomain simulations and experimental optical mapping experiments have shown agreement with predictions of SI curves for different polarities of applied stimulus (anodal or cathodal) (Kandel and Roth, 2013). SI curves are produced by applying a premature *S*_2_ stimulus of a known strength to the tissue at a given instant of refractoriness, i.e., at a specific time following the *S*_1_ paced beat. The minimum strength required to elicit propagation is then recorded, and a new timing following the *S*_1_ beat probed. Here, we extend the concept of the SI curve from unipolar to field stimulation applied to specific anatomical features. VEs from field stimulation are symmetric with respect to the polarity of the stimulus—in effect, polarities are inverted with respect to a swapping of the field direction. This means that, in contrast to the results from unipolar stimulation, SI curves from field stimulation are invariant with respect to change in sign of the stimulating electrode and, thus, the concept of “anodal” or “cathodal” stimulus does not exist for field stimulation of isolated structures.

### 2.2 Computational Modeling

#### 2.2.1 Governing Equations

The tissue is modeled using the bidomain equations for cardiac electrodynamics (Henriquez, 1992). These may be written as

(1) $$\begin{array}{ll} \nabla \cdot \left(\sigma_{i}\nabla \phi_{i}\right) & =\beta I_{m},\\ \nabla \cdot \left(\sigma_{e}\nabla \phi_{e}\right) & =-\beta I_{m},\\ I_{m} & =C_{m}\frac{\partial V_{m}}{\partial t}+I_{\text{ion}}\left(V_{m},\eta \right)-I_{s},\\ \sigma_{b}\nabla^{2}\phi_{b} & =0 \end{array}$$

where *ϕ_i_* and *ϕ_e_* are the intra- and extracellular potentials, *V_m_* = *ϕ_i_* − *ϕ_e_* is the transmembrane potential, ***σ****_i_* and ***σ****_e_* are the intra- and extracellular conductivity tensors, *σ_b_* is the bath conductivity, *ϕ_b_* is the potential field in the bath, *β* is the membrane surface area to volume ratio, *I_m_* is the transmembrane current density, *I_s_* is the transmembrane current stimulus, *C_m_* is the membrane capacitance per unit area, and *I_ion_* is the membrane ionic current density, as a function of the transmembrane potential *V_m_* and the vector of state variables ***η***. As described in Roth (1991), the boundary conditions (BCs) imposed on equation (1) are motivated by physical arguments and ensure that there is no flux of the intracellular current across the tissue boundary, and that the extracellular and bath potentials are continuous at the boundary of the tissue $\partial \Omega =\partial \Omega_{t}\cup \partial \Omega_{\mathrm{tb}}$. The subscript *t* represents tissue and subscript *tb* represents the tissue–bath boundary, so that the BCs are

(2) $$\begin{array}{ll} \vec{n}\cdot \left(\sigma_{i}\nabla \phi_{i}\right) & =0,\;\partial \Omega_{t},\Omega_{\mathrm{tb}}\;\\ \vec{n}\cdot \left(\sigma_{e}\nabla \phi_{e}\right) & =\sigma_{b}\vec{n}\cdot \nabla \phi_{b},\;\partial \Omega_{\mathrm{tb}}, \end{array}$$

where $\vec{n}$ is unit-normal to the heart tissue surface. In addition, no-flux conditions are applied to the boundary of the bath space.

#### 2.2.2 Anatomical Geometries

**Figure 1:**
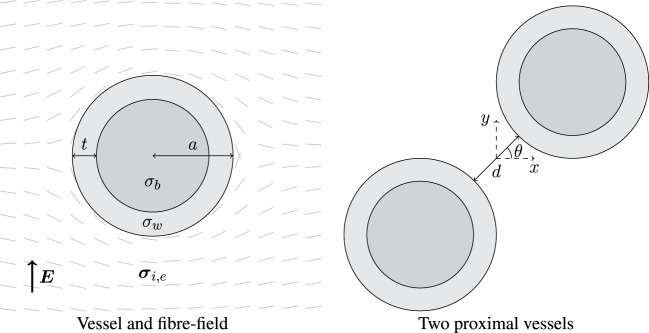
**Left: schematic of a blood vessel surrounded by myocardium, with fibers smoothly circumnavigating the vessel**. The vessel wall has thickness *t* and conductivity *σ_w_*, and the blood inside the vessel has conductivity *σ_b_*. The outer radius of the vessel is *a*, and the surrounding myocardium has anisotropic conductivity in the intra- (***σ****_i_*) and extracellular (***σ****_e_*) space. Right: two identical blood vessels proximal to one another separated by spacing *d* and oriented by angle *θ*; the origin is situated at the mid-point between the two vessel centers.

Two simplified geometries were considered: a vessel in a semi-infinite medium and two proximal vessels in a semi-infinite medium, as shown in Figure 1. The models were chosen to represent anatomical observations in an idealized way. In each case, the fiber field was always tangent to the surfaces of, and smoothly circumnavigated, the vessels as observed in histological analysis (Gibb et al., 2009); this was achieved by taking the unit-vector field of the gradient of the potential field created between two electrodes on the left and right hand side of the tissue domain, and applying a no-flux boundary condition on vessel surfaces and the upper and lower boundaries of the tissue (Bishop et al., 2010a; Bayer et al., 2012). In Figure 1, the left panel shows the smoothly varying fiber field around a single blood vessel, parameterized by its outer radius *a* and wall thickness *t*. The wall thickness was chosen to be a non-linear function of the vessel radius through (Podesser et al., 1998)

(3) $$t=3.87a^{0.63},$$

corresponding to experimental observations of the human coronary vasculature. Two values of radius *a* were chosen: 0.5 and 2.0 mm as these lie inside the range, and near the lower and upper bounds, of observed arterial radii (Podesser et al., 1998). As arteries and veins tend to be located proximal to one another, the effects of the superposition (Hörning et al., 2010) of VEs from proximal vessels were investigated by varying the angle *θ* between two vessels (middle panel, Figure 1); we chose to align them next to each other (*θ* = 0), offset them by *θ* = *π*/4 and align them above one another *θ* = *π*/2. In each case, the minimum distance between vessel radii was *d*.

**Figure 2:**
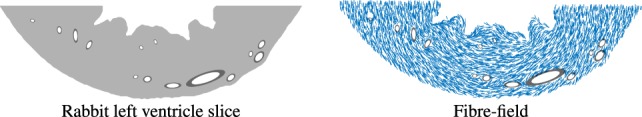
**Left: a slice from a high-resolution MRI image of the rabbit left-ventricle**. Right: the resulting fiber field from the Laplace-solve, under-sampled for clarity.

A more realistic geometry was constructed from a high-resolution MRI scan of the rabbit ventricles (Bishop et al., 2010b), in which blood vessel cavities were resolved. A cross-sectional slice was taken in the long axis and cropped to show a sector of the left ventricular wall, and the image then re-scaled to make the thickness of the left ventricular wall approximately 15 mm, similar to the human left ventricular wall thickness. Minimum bounding ellipses were fitted around the blood vessel cavities, giving two values for the vessel radius corresponding to the semi-major *r*_1_ and semi-minor *r*_2_ axes of the bounding ellipse. The thickness *t* of the vessel wall was assumed to vary as a linear function of the radius using

(4) $$t\left(\psi \right)=\mathrm{kr}\left(\psi \right)=k\frac{r_{1}r_{2}}{\sqrt{{\left(r_{1}\cos\left(\psi \right)\right)}^{2}+{\left(r_{2}\sin\left(\psi \right)\right)}^{2}}},$$

where *k* ≈ 0.18, calculated from the mean wall thickness for (circular) vessels with radii of 0.5 and 2.0 mm, and *ψ* ∈ [0, 2*π*] is the polar angle. The outer radius of the vessel was thus taken to be *a*(*ψ*) = (1 + *k*)*r*(*ψ*). Figure 2 (left panel) shows the geometry produced. Varying fiber architecture was then assigned (right panel, Figure 2) using the same method as the simplified vessels.

The geometries in Figures 1 and 2 were discretized with linear triangular finite elements, with an average edge length of approximately 75 *μ*m, and internal boundaries (vessel and vessel wall surfaces) were highly refined; the average edge length of elements forming the *a* = 0.5 and 2.0 mm vessel cavities was approximately 12 and 22 *μ*m, respectively.

#### 2.2.3 Electrophysiological Tissue Representation

Ionic cellular dynamics were represented by a human ventricular cell model (ten Tusscher and Panfilov, 2006), which has been used in many tissue-level human computational modeling studies (Ashikaga et al., 2013; Rantner et al., 2013a; Arevalo et al., 2016). To reproduce the asymmetry of the membrane response to strong shocks delivered during the plateau phase of the action potential, the cell model was further augmented (DeBruin and Krassowska, 1998; Ashihara and Trayanova, 2004) with an electroporation current and a hypothetical potassium current that activates at larger depolarizations of >160 mV; these additional currents are simply summed with the *I_ion_* term in equation (1) and do not require any modification to the cell model ordinary differential equation system (ten Tusscher and Panfilov, 2006).

The domains were assigned physiologically realistic values of conductivity. In the myocardium, the intra- and extracellular conductivity tensors ***σ****_i_* = (*σ_i,l_,σ_i,t_*) = (0.17, 0.019) S/m and ***σ****_e_* = (*σ_e,l_,σ_e,t_*) = (0.62, 0.24) S/m were given experimentally measured values (Clerc, 1976) for the directions longitudinal (subscript *l*) and transverse (subscript *t*) to the local fiber orientation, giving representative conduction velocities in resting tissue of approximately 61 (longitudinal) and 22 (transverse) cm/s. The vessel walls were assigned the experimentally measured (isotropic) value of *σ_w_* = 0.01 S/m (Bishop et al., 2010a), and the blood inside the vessels and surrounding the myocardium was assigned the value of *σ_b_* = 1.0 S/m (Visser, 1989).

#### 2.2.4 Computational Considerations

The finite element solver CARP (Cardiac Arrhythmia Research Package) (Vigmond et al., 2003) was used to solve the bidomain equations (1) and (2). The prescribed time step was set to 5 *μ*s; a value known to be stable and accurate (Cooper et al., 2016) for the *ten-Tusscher* (ten Tusscher and Panfilov, 2006) human ventricular model, and sufficiently low to ensure the ramp function for the shock was resolved smoothly.

### 2.3 Stimulation Protocol

#### 2.3.1 Tissue Preconditioning

Initially, the tissue was pre-paced at the single-cell level using a basic cycle length of 300 ms, for 100 beats. The saved state variables were then applied to every reaction source term in the tissue-level model.

#### 2.3.2 Strength–Interval Computation

In order to avoid introducing spatial gradients in refractoriness within the tissue, the entire tissue was uniformly excited using a 2 ms *S*_1_ transmembrane stimulus. The state of the entire bidomain simulation was then saved at 5 ms time intervals from 250 to 350 ms after the last *S*_1_ stimulus; the time values were chosen as they span the relatively refractory to the fully excitable phases of the action potential. These saved states are the *x*-values of the SI curve.

For each saved time increment, the minimum strength of an *S*_2_ field stimulus required to elicit wave propagation from the heterogeneities present in the tissue (the vessels) was computed. This was performed using a simple bisection scheme with an upper bound of 10 V/cm, using the criterion that an excitation wavefront was found to be propagating somewhere in the tissue 30 ms after the termination of the stimulus with a minimum peak transmembrane potential (action potential amplitude) of −10 mV (a value found to be reasonable from visual inspection of the shock-induced wavefronts). A tolerance on the bisection scheme of <0.1 V/cm for the *S*_2_ strength was used. The *S*_2_ field stimuli were imposed by applying dissimilar Dirichlet boundary conditions, in the extracellular space, to the upper and lower surfaces of the tissue domains. These minimum field strengths then constitute the *y*-values of the SI curve.

For each of the idealized geometries considered, excitation from surface VEs on the depolarized surface of the tissue domains was avoided by imposing a layer of unexcitable (passive) tissue with sufficient thickness (approximately six times the transverse space-constant) to attenuate the surface VE amplitude sufficiently, and the boundaries were located far from the vessel to negate boundary effects. Such an approach did not affect the strength or distribution of the electric field around the anatomical structures considered.

## 3 Results

### 3.1 Anisotropic Tissue

**Figure 3:**
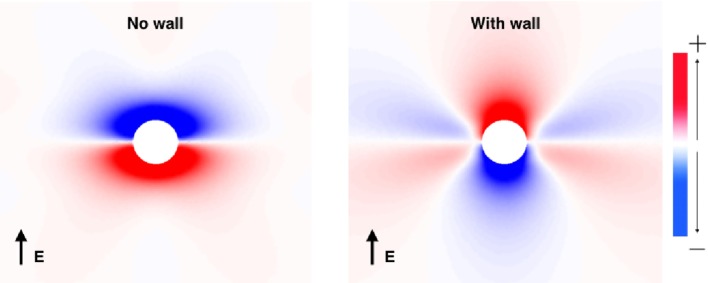
**The VE patterns around a blood vessel with (*right*) and without (*left*) the presence of a vessel wall during field stimulation**. Red colors show depolarization, and blue colors show hyperpolarization.

Prior to investigating the SI curves for the vessel configurations shown in Figure 1, we first show how the VE patterns change when the low-conductivity vessel wall is included in the bidomain model. This was done by solving the bidomain equations with a passive membrane (*I_ion_* = *V_m_*/*R_m_*) until steady state is achieved. The steady-state VE patterns for a vessel with and without vessel walls are shown in Figure 3. Figure 3 clearly illustrates that the VE pattern is changed by the presence of the low-conductivity vessel wall. The most significant difference is the swapping of the polarity at the upper and lower surfaces of the vessel, an effect observed previously (Bishop et al., 2010a, 2012). A secondary effect of the presence of a vessel wall is to increase the magnitude of VEs which form on either sides of the vessel due to the anisotropic conductivity ratios. The physical reason for these two effects is that: (a) in the case of no vessel wall, boundary VEs from current transiting the extracellular-to-bath-to-extracellular spaces dominate, as current takes the path of least resistance through the vessel cavity, and (b) in the case of a vessel wall, the insulating vessel wall changes the path of least resistance from through the vessel cavity to around the vessel cavity, thus VEs from dissimilar anisotropy ratios dominate, and boundary VEs are diminished.

**Figure 4:**
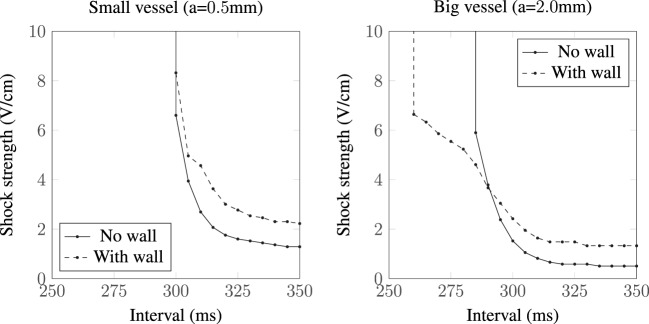
**SI curves for small and large vessels with and without vessel walls**.

The different VE patterns shown above in Figure 3 act to change the active membrane response, as shown in the SI curves in Figure 4. Figure 4 shows that for late diastolic intervals (DIs) of greater than ≈315 ms, the SI curve is approximately linear and near the asymptotic, minimum, value. At such late DIs, the mechanism of tissue capture is via make excitation, with wave propagation initiating from the vessel almost immediately as the *S*_2_ stimulus is applied. The shock strength at late DIs for vessels without vessel walls is approximately half that compared to the case with a vessel wall, and the shock strength required to elicit excitation from larger vessels is lower than that required for smaller vessels, consistent with the literature (Pumir and Krinsky, 1999; Hörning et al., 2010; Luther et al., 2012). For all vessels (in Figure 4), the shock strength monotonically increases with decreasing DI. At early DIs, the shock strength required to elicit propagation for the larger vessel with a vessel wall is lower than that without a vessel wall, a direct consequence of the altered VE pattern around the vessel from the influence of the insulating vessel wall. We include a vessel wall in the rest of the results in this manuscript, as the presence of the vessel wall significantly alters the SI curves.

**Figure 5:**
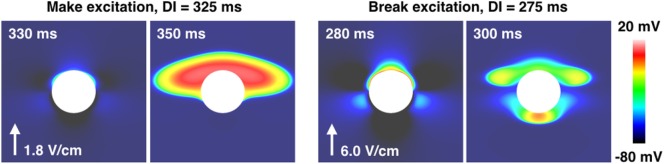
**The propagation patterns created by the *a* = 2.0 mm vessel with the vessel wall, for early (right panel) and late (left panel) DIs, resulting in make and break excitations**. The shock strength is quoted next to the arrow showing the field direction in each case.

Figure 5 shows the types of propagation patterns created by the VEs around the large (*a* = 2.0 mm) blood vessel, with a vessel wall, at different DIs and shock strengths causing make and break excitation patterns. Break excitation (right panel in Figure 5) shows how the depolarized regions rapidly diffuse into the now-excitable hyperpolarized regions, causing waves to propagate through these previously hyperpolarized regions. Without the close proximity of de- and hyperpolarized VE regions, the mechanism of break excitation would not occur.

It should be noted that we investigated the SI response (results not shown) in the absence of the electroporation currents (Ashihara and Trayanova, 2004) and found similar behavior as reported in Bray and Roth (1997); the addition of the electroporation currents acts to reduce the shock strength required to elicit propagation from VEs.

#### 3.1.1 Influence of Conductivity Variations

**Figure 6:**
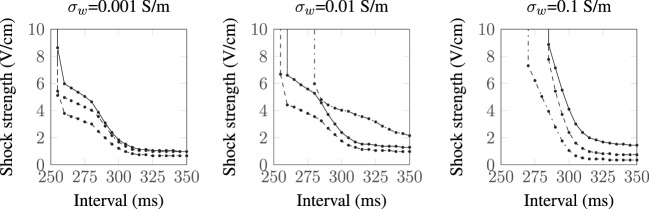
**SI curves for the *a* = 2.0 mm vessel with different tissue and vessel wall conductivities: 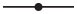 Clerc (1976); 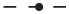 Roberts et al. (1979); and 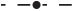 Roberts and Scher (1982)**.

As there is some uncertainty regarding the conductivities of myocardium and the vessel wall, we recomputed the SI curves for the largest vessel (*a* = 2.0 mm) with other different sets of experimentally measured (Roberts et al., 1979; Roberts and Scher, 1982) tissue conductivities (Roth, 1997) and varied the vessel wall conductivity by two orders of magnitude around the experimentally measured value of *σ_w_* = 0.01 S/m (Bishop et al., 2010a). The tissue conductivities used are summarized in Table 1, and the resulting SI curves are shown in Figure 6. Figure 6 shows that, for the lowest vessel wall conductivity (left panel), the different tissue conductivities have little influence on the shape of the SI curve. With the vessel wall conductivity set at its experimentally measured value (middle panel), the third tissue conductivity set (Roberts and Scher, 1982) causes the SI curve to diverge appreciably from the rest of the curves at early DIs. At the highest vessel wall conductivity (right panel), the SI curves for each tissue conductivity set are appreciably different from the other two panels and appear similar to the solid curve shown in Figure 4 (right panel), which corresponds to the case of a vessel without a vessel wall. This is to be expected as the higher wall conductivity is more similar to the blood conductivity in the vessel cavity. Overall, the different tissue conductivities have little effect on the shape of the computed SI curves. The value of the vessel wall conductivity tends to dictate the shape of the SI curve; this is because the induced VE patterns are different when the conductivity of the vessel wall is much smaller than the conductivity of the vessel cavity (blood)—see Figure 3.

**Table 1:** **Various published tissue conductivities (in S/m)**.

Source	*σ_il_*	*σ_it_*	*σ_el_*	*σ_et_*
Nominal (Clerc, 1976)	0.170	0.019	0.620	0.240
Roberts et al. (1979)	0.280	0.026	0.220	0.130
Roberts and Scher (1982)	0.340	0.060	0.120	0.080

*Here, “nominal” refers to the conductivity set used in the rest of this work*.

### 3.2 Modulation of SI Curve Response due to Superposition Effect of Neighboring Vessels

**Figure 7:**
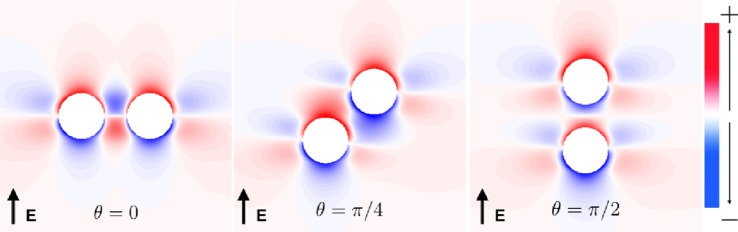
**Steady-state VE patterns for *a* = 2 mm vessels separated by distance *d* = a**.

The vessels comprising the coronary vasculature are often colocated, with veins following the same tracts as arteries in vessel pairs. The proximity and orientation of two blood vessels alters the resultant VE pattern produced because the fiber architecture is different for two proximal vessels, compared to that for one vessel, and also because the potential fields in the intracellular and extracellular spaces are linear and obey the principle of superposition (Hörning et al., 2010). In the case where the vessels are aligned perpendicular to the applied field (left panel, Figure 7), the positive and negative VEs between the two vessels are amplified. When the vessels are oriented at *π*/4 from one another (middle panel, Figure 7), the positive VE at the top of the left vessel and the negative VE at the bottom of the right vessel are amplified. When the vessels are arranged in the direction of the applied field (right panel, Figure 7), the positive and negative VEs between the two vessels are reduced in magnitude.

**Figure 8:**
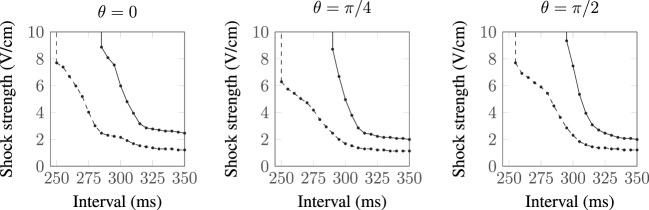
**SI curves for two proximal vessels, separated by spacing *d* = *a* and oriented relative to one another by angle *θ* (as described in Figure 1)**. *a* = 0.5 mm: 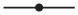, *a* = 2.0 mm: 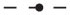.

The complex non-linear dynamics of the membrane response to these VEs of different patterns results in different SI curves for these vessels, as shown in Figure 8. For each of the vessel alignments, the SI curve is similar for late DIs of between 325 and 350 ms; this is because make excitation dominates at late DIs, which is relatively independent of the complex VE patterns produced by the different vessel configurations. At early DIs of less then 300 ms, the different VE patterns result in different SI curves; however, the differences are minimal and each vessel alignment gives a similar response to the single vessel (with a vessel wall), shown in Figure 4. The *θ* = *π*/4 alignment requires the lowest shock strength to elicit propagation overall due to its configuration leading to the proximity of the strongest VE polarizations.

### 3.3 VE Induced Propagation from a Realistic Ventricular Slice

**Figure 9:**
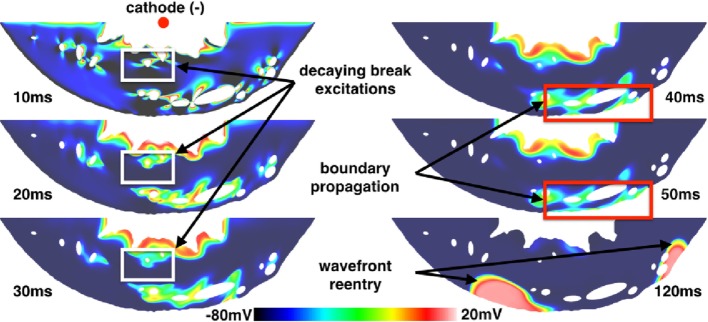
**VE distributions, break excitation decay (highlighted in white rectangles), and propagated graded responses (highlighted in red rectangles) from a 10 ms duration shock of 10 V/cm applied at a DI of 290 ms in the realistic ventricular slice**. The times refer to the time elapsed after the initiation of the shock.

The ventricular slice geometry was pre-paced at the tissue level in a similar manner as for the isolated blood vessels; however, the SI curve was not computed. Instead, shocks were applied from anodal and cathodal electrodes in the bath space in the middle of the left ventricular cavity to a ground in the bath space surrounding the epicardium, resulting in an approximately transmural field. Shocks of 10 V/cm (computed via the minimum Euclidean distance from points on the surface of the cathode to the ground) were applied at DIs of between 290 and 350 ms after the previous stimulus. Regardless of the polarity of the electrode, the same type of make excitations were observed at late DIs (between 300 and 350 ms) from the transmural vessels and the same type of break excitations were observed at early DIs (between 290 and 300 ms), as with the idealized vessel geometries considered previously. As with the idealized vessel geometries, break excitations either continued to propagate or decay (as shown in Figure 9) depending on the DI at which the shock was applied.

However, a different mode of break excitation in the form of propagated graded responses (Trayanova and Rantner, 2003; Trayanova et al., 2003) attached to the subepicardial layer was observed around vessels proximal to the epicardium. Figure 9 shows snapshots of the transmembrane potential in the ventricular slice following the application of the shock. These boundary waves were caused by VE depolarizations around subepicardial vessels propagating into VE hyperpolarized regions which intersected the epicardium. The boundary propagation waves had peak transmembrane potentials of approximately −10 mV and action potential durations of approximately 35 ms; values significantly below the normal electrophysiological characteristics of the (ten Tusscher and Panfilov, 2006) cell model and similar to the propagating unstable wavelets described in Boyle et al. (2012). Approximately 120 ms after the shock was applied, these boundary propagation wavefronts re-entered and excited the entire tissue. By saving the cell model state variables (DeBruin and Krassowska, 1998; Ashihara and Trayanova, 2004; ten Tusscher and Panfilov, 2006) from nodes inside the boundary propagation waves and continuing the integration at the single-cell level, we determined that the short action potential durations and low transmembrane amplitudes of these waves were a direct consequence of electrotonic coupling and not an electrophysiological effect of the cell model itself. In contrast to the boundary propagation waves, single-cell action potentials had durations of approximately 200 ms (short in contrast to the normal human ventricular duration) and peak transmembrane potentials of approximately 20 mV.

Note that the transmembrane potential at the epicardium had equalized (from its shock-induced hyperpolarization) with the surrounding transmembrane potential when the boundary propagation was observed. At higher shock strengths, however, break excitations from vessel VEs reached the epicardium earlier and resulted in more rapid boundary propagation; this was due to the latent hyperpolarization from the shock-induced VEs. During anodal shocks, the inverted VE pattern gave rise to break excitations next to the endocardial surface from adjacent hyperpolarized areas; however, these boundary propagation waves rarely gave rise to bulk activation as they were often extinguished after collision with regions of prolonged refractoriness (due to depolarized VE regions) or failed to propagate along the relatively more curved endocardial boundary (trabeculation).

### 3.4 Isotropic Tissue

Some insight on the origins of the VEs elicited by blood vessels in isotropic tissue may be gained by using an analytical approach. Unlike anisotropic tissue where VEs may form in the tissue in response to conductivity anisotropy, VEs in isotropic tissue may originate only at tissue surfaces, where current enters or exits the bidomain. At steady state, the governing equations for isotropic tissue simplify to

(5) $$\begin{array}{ll} \nabla^{2}V_{m}-V_{m}∕\lambda^{2} & =0,\;\in \Omega_{t}\\ \left(\sigma_{i}+\sigma_{e}\right)\nabla^{2}\phi_{e} & =0,\;\in \Omega_{t}\\ \sigma_{b}\nabla^{2}\phi_{b} & =0,\;\in \Omega_{b}, \end{array}$$

where $\lambda^{2}=R_{m}\sigma_{i}\sigma_{e}∕\left(\beta \left(\sigma_{i}+\sigma_{e}\right)\right)$ is the space constant (here, *R_m_* is the membrane resistance). Equation (5) assume that perturbations to ∇*ϕ_e_* from the induced transmembrane potential (in response to field stimulation) are small, such that they may be neglected, and assumes a parallel combination of the intra- and extracellular isotropic conductivities for Laplace’s equation in the extracellular space (Sobie et al., 1997; Jolley et al., 2008). Equation (5) may be solved by separation of variables as in Pumir and Krinsky (1999), except here instead of assuming a constant extracellular field for ∇*ϕ_e_* (Pumir and Krinsky, 1999); we first solve Laplace’s equation for *ϕ_e_* to satisfy the electrostatic boundary conditions at the interfaces of the vessel cavity and vessel wall, and vessel wall and tissue. Assuming the far-field stimulus, of magnitude *E*_0_, is aligned with the *y*-axis $\left(\text{E}=E_{0}\vec{y}\right)$ gives, in polar coordinates:

(6) $$V_{m}\left(r,\theta \right)=CE_{0}\lambda \frac{K_{1}\left(r∕\lambda \right)}{{K\prime }_{1}\left(a∕\lambda \right)}\sin\left(\theta \right),$$

where *K_ν_* is the modified Bessel equation of order *ν*, of the second kind, and *C* is a scaling factor which comes from the solution of Laplace’s equation for the particular geometry considered:

(7) $$\begin{array}{l} C=2\left(\sigma_{b}\sigma_{w}\left(a^{2}+{a\prime }^{2}\right)+\sigma_{w}^{2}\left(a^{2}-{a\prime }^{2}\right)\right)/\;\left[\;\right.\left(\sigma_{i}+\sigma_{e}\right)\;\left[\sigma_{w}\left(a^{2}+{a\prime }^{2}\right)+\sigma_{b}\left(a^{2}-{a\prime }^{2}\right)\right]\;+\sigma_{b}\sigma_{w}\left(a^{2}+{a\prime }^{2}\right)+\sigma_{w}^{2}\left(a^{2}-{a\prime }^{2}\right)]\;, \end{array}$$

where *a*′ = *a* − *t*. Assuming that the electric field is constant around the vessel implies that *C* = 1, giving the solution in Pumir and Krinsky (1999).

Substituting the transverse values for the intra- and extracellular conductivities (*σ_it_* = 0.019, *σ_et_* = 0.24 S/m) (Clerc, 1976) (approximating an out-of-plane fiber field parallel to the vessel axis), the blood (*σ_b_* = 1.0 S/m) and vessel wall conductivities (*σ_w_* = 0.01 S/m), into *C* and using the analytical expression for the wall thickness [equation (3) from Podesser et al. (1998)] gives *C* ≈ 0.15 and *C* ≈ 0.25 for vessels of radii *a* = 0.5 and *a* = 2.0 mm, respectively. In other words, the low-conductivity blood vessel wall reduces the current flux through the vessel cavity, significantly lowering the VE induced by the vessel in response to field stimulation. In the case of no vessel wall, the scaling factor becomes independent of the vessel size and simplifies to *C* = 2*σ_b_*/(*σ_b_* + *σ_i_* + *σ_e_*) ≈ 1.59.

**Figure 10:**
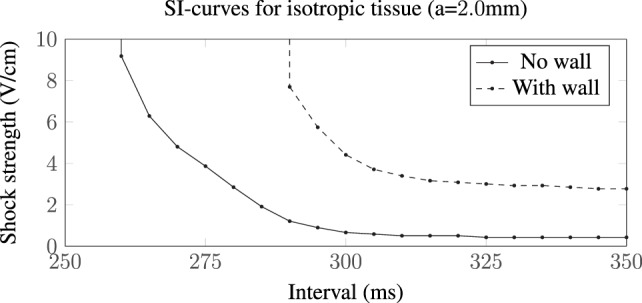
**SI curves for the *a* = 2.0 mm vessel in isotropic tissue**. The presence of a low-conductivity vessel wall increases the field strength required to elicit wave propagation by a factor of approximately 6.44 compared with the vessel without a vessel wall. In these computations, we used the transverse conductivity values; *σ_it_* = 0.019, *σ_et_* = 0.24 S/m (Clerc, 1976), approximating an out-of-plane fiber field.

The SI curve for a vessel of radius *a* = 2.0 mm in isotropic tissue is shown in Figure 10. The ratio in the field strengths required to elicit excitation, from the vessels with and without a vessel wall, is approximately 6.44 at an interval of 350 ms, a value which is approximately constant within the interval range of validity (290–350 ms). This is close to the ratio of the scaling factors, for the *a* = 2.0 mm vessel with and without a vessel wall, of 1.59/0.25 ≈ 6.36, suggesting that, in the case of isotropic tissue, the scaling factor is proportional to the minimum field strength required for VE induced excitation from the blood vessels. In isotropic tissue, the VE pattern is identical for vessels with and without vessel walls (albeit with different magnitudes)—these patterns are shown and discussed in detail in Pumir and Krinsky (1999) and Bittihn et al. (2012).

## 4 Discussion

In this work, idealized representations of blood vessels were used to investigate how the vessel-induced VEs influenced the electrophysiology of the surrounding tissue in different states of refractoriness upon field stimulation.

### 4.1 VEs around Idealized Blood Vessels and SI Curves

SI curves have been previously computed for unipolar stimulation both experimentally (Dekker, 1970; Sidorov et al., 2005) and in numerical bidomain simulations (Kandel and Roth, 2013, 2014). In general, the same mechanisms of make and break excitations, and the same overall morphology of the curves were observed from field stimulation of blood vessels, as have been observed (numerically and experimentally) in unipolar stimulation of myocardium (Sidorov et al., 2005; Kandel and Roth, 2013, 2014). Direct experimental validation of the VE patterns predicted by our simulations is challenging, due to the fact that vessels are inherently intramural. Thus, the close agreement in overall excitations mechanisms and SI curve morphologies with respect to previous experimental unipolar stimulation studies represents an important means of comparison. It should be noted that in both cases, break excitation occurs due to the co-location of de- and hyperpolarized VEs induced by the stimulus.

#### 4.1.1 Excitation Behavior at Late DIs—“Make Excitations”

The VE pattern created upon field stimulation depends upon whether the blood vessel wall is resolved in the geometry, as shown in Figure 3. At late DIs, the tissue surrounding the vessel is relatively excitable and VE depolarizations of a sufficient magnitude cause wave propagation (see Figure 5, left panel).

Without a vessel wall, the shock strengths required for make excitations are lower than the case with the vessel wall, as shown in Figure 3. This is because current takes the path of least resistance: with the insulating vessel wall, current is shielded from traversing the vessel cavity (lowering the magnitude of the boundary flux VE) and instead prefers to travel around the vessel (raising the magnitude of the VE contribution from the varying fiber architecture). In addition, the shock strength required to elicit make propagations was shown to be lower for larger vessels (with and without vessel walls) in agreement with the literature (Pumir and Krinsky, 1999; Luther et al., 2012).

The effect of the superposition of VEs and different fiber architecture around vessels proximal to one another is minimal at late DIs (see Figure 8) as the complicated VE pattern has relatively little influence when the surrounding tissue is relatively excitable.

#### 4.1.2 Excitation Behavior at Early DIs—“Break Excitations”

With decreasing DI, the SI curve monotonically increases, and the larger vessels display, in general (see Figures 4 and 8), three different slopes; shallow from 350 to 300 ms, sharp from 300 to 275 ms, and then decreasing slightly from 275 to 250 ms. These different slopes in the SI curves for the larger vessels correspond roughly with the shape of the repolarizing action potential (*V_m_*(*t*)). In the case of the large blood vessel, wave propagation is possible at lower strengths when the vessel wall is resolved (see Figure 4, right panel). This is because the VE pattern changes in the presence of an insulating vessel wall (see Figure 3), such that VEs of opposite polarity are relatively proximal to one another far away (Takagi et al., 2004) from the vessel cavity, which acts as a boundary to diffusion. The mode of excitation at early DIs is via break excitation, where, at the end of the shock, the depolarized region rapidly diffuses into the hyperpolarized region (which was in the refractory phase at the beginning of the shock, but has since been made excitable by VE hyperpolarization) and initiates wave propagation. This type of propagation may decay or may continue to propagate into the surrounding tissue (depending on its refractoriness). The same phenomenon (wave propagation from a vessel with a vessel wall at early DI) may occur for the smaller blood vessel; however, this was outside the parameter space investigated in this work.

When vessels were arranged close to one another, the altered VE patterns acted to change the SI curve at early DIs; however, the changes were not major; the same monotonic increase in shock strength at earlier DIs occurred. When vessels were arranged at *θ* = *π*/4 to one another, the strength required to elicit break excitation was lower overall; with this configuration, the two largest magnitude VEs were immediately next to one another.

### 4.2 VEs around Other Heterogeneities

VEs may form around any heterogeneities, and the non-dimensional magnitude $\left(\left|V_{m}∕\left(E_{0}\lambda \right)\right|\right)$ of such VEs is dependent on the conductivity ratio of the heterogeneity and the surrounding tissue, and the characteristic length scale of the heterogeneity with respect to the local field direction ***E***. For example, it is shown (Pumir et al., 2007) that, in isotropic tissue, elliptical heterogeneities oriented with their semi-major axes parallel to the local field require a stronger stimulus to elicit wave propagation than those oriented with their semi-minor axis parallel to the applied field. However, regardless of the geometry of the heterogeneity, or the direction of the local electric field, in isotropic tissue, the maximum non-dimensional VE is scaled by the conductivity ratio [discussed in equations (6) and (7)]. This does not hold, however, in anisotropic tissue, rendering it difficult to compare the magnitudes of VEs created by cylindrical blood vessels with other heterogeneities, such as fibrosis or sheets. In general, however, for heterogeneities in isotropic tissue with a non-zero conductivity ratio, the magnitude of the non-dimensional VE is approximately constant (and approximately equal to the conductivity ratio), provided the characteristic length scale is much bigger than the space constant (Pumir and Krinsky, 1999). The conductivity of regions of fibrosis is known to be much smaller than for healthy myocardium, so it is likely that the VEs elicited by these fibrosis heterogeneities will be dominated by anisotropy effects.

### 4.3 Relevance for Low-Energy Defibrillation Protocols

Previous experimental works highlighting the potential utility of low-energy defibrillation protocols (Fenton et al., 2009; Luther et al., 2012) have suggested the importance of the VEs formed around the coronary vasculature in providing a distributed source of wavefronts that facilitate arrhythmia termination. A consideration not addressed by these earlier works is the specific mechanism by which wavefront propagation is elicited from the vessel by the applied field with respect to the degree of refractoriness of the tissue.

Here, we have shown that break excitations may occur around blood vessels in otherwise refractory tissue. The ability to elicit wavefronts from relatively refractory tissue and not just from recovered diastolic tissue could therefore play a crucial role in optimizing low-energy defibrillation methods (Fenton et al., 2009; Janardhan et al., 2012; Luther et al., 2012; Rantner et al., 2013b) for which removing both excitable and soon to be excitable tissues is the main underlying goal. In the context of low-energy defibrillation, this may be especially relevant considering that the timing of the defibrillation shock, with respect to the time evolution of the excitable volume (Rantner et al., 2013b), is known to influence the defibrillation efficacy. However, an important finding from our study is that the field strength at which break excitations occur appears to be *above* that of low-voltage regimes, which typically quote field strengths <1 V/cm (Fenton et al., 2009; Luther et al., 2012; Rantner et al., 2013b). Therefore, our analysis suggests that such a mechanism may only be important in protocols that involve relatively higher strength shocks.

In this work, it was assumed that the tissue around the vessel repolarized simultaneously; however, in reality, a repolarization gradient, aligned with the propagation vector of the previous wave, will exist around the vessel. In sinus rhythm, the excitation generally propagates from the endocardium to the epicardium, producing a typical repolarization gradient of approximately 4.5 ms/mm—a value known to be sufficient to cause unidirection propagation from extrasystoles (Laurita and Rosenbaum, 2000) in guinea-pig hearts. It is however difficult to draw any conclusions regarding the arrhythmogenicity of extrasystoles originating from VEs around blood vessels; the induced VEs themselves will perturb the repolarization field and, in context, shocks will typically only be applied during fibrillation when the wave dynamics are chaotic and, by definition, not in sinus rhythm. The results from this work are mainly applicable to regions of tissue with small repolarization gradients, which may exist in regions around the myocardium during fibrillation.

### 4.4 A Note on Shock Strengths

**Figure 11:**
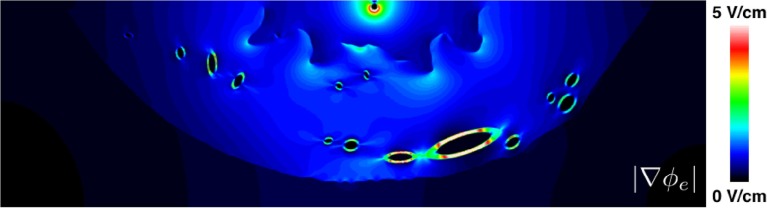
**The spatial variation of the magnitude of the electric field around the realistic ventricular slice geometry**. Note that the color bar maximum was lowered from its theoretical maximum (10 V/cm) to 5 V/cm in order to highlight the regions of field concentration.

In this work, and in much of the literature on defibrillation, the shock strength is quoted as the potential difference (Δ*ϕ_e_*) between the shock electrodes divided by the distance between them (*l*). A single measure of shock strength is a crude approximation when the conductivity in the domain is not constant, as is generally the case, and when one of the shocking electrodes is a catheter. The magnitude of the stimulating electric field, $|\text{E}|=|\nabla \phi_{e}|$, which measures the shock strength as a function of position, may vary significantly even in simple or idealized geometries such as considered in this work. To highlight this point, see Figure 11 which shows the heterogeneous nature of $|\nabla \phi_{e}|$ for the $\left(\Delta \phi_{e}∕l\right)\;\approx \;10\;V∕\text{cm}$ shock, which elicited the boundary wave propagation in the realistic slice geometry. Figure 11 shows how the field strength decays rapidly (approximately as 1/*r^d^* ^− 1^, where *d* is the number of dimensions) away from the point cathode and concentrates around the endocardial grooves [which, incidentally, is the reason for the preferential wave propagation from these regions as discussed in Rantner et al. (2013b)] and low-conductivity vessel walls. Thus, we highlight that quoted shock strengths for specific protocols may differ significantly from the localized field strengths sensed in specific regions of myocardium.

### 4.5 Boundary Propagation Mediated by Subepicardial Vessels

Subepicardial boundary propagation was observed to occur at early DIs in the image-derived ventricular slice geometry (see Figure 9). The phenomenon, observed after shocks in the relatively refractory period may represent a mode of shock-induced arrhythmogenesis, or an additional mechanism responsible for the earliest post-shock activations after the isoelectric window (Trayanova, 2007; Ashihara et al., 2008; Constantino et al., 2010). We hypothesize that the low transmembrane potential amplitude and short action potential duration of the propagating boundary wave, in addition to the shallow depth in which it propagates, may have prohibited its *in vitro* observation using voltage sensitive dyes in optical mapping experiments due to depth-averaging effects (Janks and Roth, 2002a,b; Bishop et al., 2007). However, it could be possible to observe this proposed mechanism using optrodes which record fluorescent signals directly from the intramural space.

It is thought that the boundary propagation waves are a form of “propagating unstable wavelets,” as described in Boyle et al. (2012). It is thought that due to the sealed boundary, current flows along the edge and accumulates longitudinally. This is just enough current to excite longitudinally but not transversely, with a partial activation of the fast sodium channel (Boyle et al., 2012). The electrotonic loading is also lower near the boundary due to the boundary condition, so a lower transmembrane current density is required to excite tissue down the concentration gradient (Kelly et al., 2013; Bishop et al., 2014). In addition, the high extracellular conductivity adjacent to the boundary (in the bath space) significantly increases the conduction velocity of the wave close to the surface (Henriquez et al., 1996, 2007; Bishop and Plank, 2011; Bishop et al., 2011). As the largest blood vessels in the coronary vasculature reside in the subepicardial layer and the magnitude of the induced VE depends on the size of the vessel, it is more likely that shocks involving an intracardiac *cathode* will lead to boundary propagation along the subepicardium, as this shock polarity leaves the epicardium hyperpolarized and excitable post-shock.

### 4.6 Implications for Computational Modeling of Field Stimulation

As shown in Figure 3 and in other works (Bishop et al., 2010a, 2012), specifically representing the insulating blood vessel wall within the computational model significantly alters the VE pattern created around the vessel, upon application of a field stimulus. The effect of resolving the blood vessel wall on the electrophysiological response of the tissue to field stimulus, as a function of the surrounding tissue refractoriness (DI) and shock strength, is shown to be significant (see Figure 4). Thus, it is important to resolve the coronary vasculature in computational bidomain models of field stimulation and to take account of the vessel wall, which was experimentally measured to have a conductivity of approximately two orders of magnitude lower than blood (Bishop et al., 2010a). The physical resolution of the blood vessel wall may however be computationally prohibitive, due to the necessarily small dimensions of the vessel wall. We propose that in more anatomically detailed 3D models, it may be possible to represent the macroscopic effect of the vessel wall by artificially lowering the conductivity of the medium inside the vessel cavity, such that the induced VE pattern is similar.

**Figure 12:**
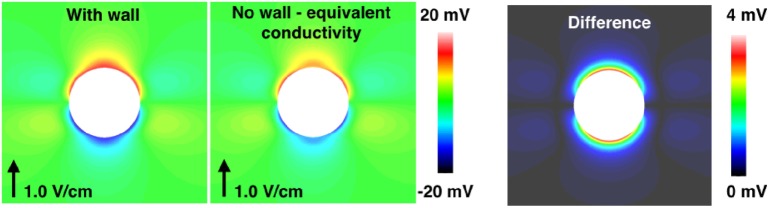
**The steady-state VE fields around a 2.0 mm radius vessel in passive anisotropic tissue: with the vessel wall resolved (left panel), and without the vessel wall resolved and the conductivity of the cavity set to the equivalent conductivity from equation (8) (middle panel)**. The magnitude of the difference in the two VE fields is shown in the right panel.

We demonstrate this in Figure 12 for the *a* = 2.0 mm vessel, where we applied a homogeneous equivalent conductivity *σ_eq_* ∈ [*σ_w_, σ_b_*] to the vessel cavity. The equivalent conductivity may be computed by solving Laplace’s equation for the potential field in an (piecewise) isotropic medium, for the vessel geometry, ensuring the current density at the vessel surface, with the equivalent homogeneous conductivity, equals that when the vessel wall and blood are resolved (i.e., at the surface of the vessel, $\sigma_{w}\vec{n}\cdot \nabla \phi =\sigma_{\mathrm{eq}}\vec{n}\cdot \nabla \phi$ where $\vec{n}$ is the unit normal vector). The equivalent conductivity is stated as

(8) $$\sigma_{\mathrm{eq}}=\frac{t^{2}\sigma_{w}\sigma_{\mathrm{bw}}+2\mathrm{at}\sigma_{w}\sigma_{\mathrm{bw}}+2a^{2}\sigma_{b}\sigma_{w}}{2\mathrm{at}\sigma_{\mathrm{bw}}-t^{2}\sigma_{\mathrm{bw}}+2a^{2}\sigma_{w}},$$

where *σ_bw_* = *σ_b_* − *σ_w_*. Equation (8) provides a good approximation in the case of isotropic tissue (results not shown), but under-predicts the VE strength in anisotropic tissue, as shown in Figure 12. It is worth noting that, when the conductivities and dimensions are substituted into equation (8), the equivalent conductivity *σ_eq_* is small (approximately two orders of magnitude lower than the minimum extracellular conductivity, for both the small and large vessels), implying that a zero-flux boundary condition for the extracellular potential at the vessel surface may provide an adequate approximation. These approaches require more investigation, however. An alternative approximation may be to apply a Robin-type boundary condition for the extracellular field across element edges/faces which constitute the blood vessel, in order to modify the extracellular current flux in a similar manner to that done for the intracellular current in Costa et al. (2014). Each of these proposed approximations requires the blood vessel surface to be spatially resolved in the computational models, however.

An additional implication of resolving the coronary vasculature is that it naturally distributes VE sources in the myocardium, causing break excitations in relatively refractory tissue which may result in boundary propagation along the hyperpolarized surface as shown in Figure 9. Without the proximity of VE depolarizations to the hyperpolarized boundary, this type of excitation may not occur. However, this mode of excitation does not require the vessel wall to be resolved *per se*.

### 4.7 Limitations

The models used in this study were two dimensional, and thus, no out-of-plane effects on the VE patterns were investigated. However, using symmetry, we have investigated the effects of a homogeneous out-of-plane fiber field in the section on isotropic tissue. The accuracy of the experimentally measured conductivity for the blood vessel wall has some uncertainty, as this has only been measured once (Bishop et al., 2010a); the vessel wall conductivity is shown to significantly influence the shape of the SI curve (see Figure 6). The *S*_1_ stimulus procedure resulted in no repolarization gradients around the vessels, which, although unphysiological, allowed us to generate SI curves which were not dependent upon initial propagation patterns. The propagating unstable wavelets have been, to the authors’ best knowledge, hitherto experimentally unobserved—thus, there is some uncertainty regarding the accuracy of these results. The effects of biphasic shocks were also not investigated in this work as here we sought to relate more closely with lower energy protocols which use monophasic shocks (Fenton et al., 2009; Janardhan et al., 2012; Luther et al., 2012; Rantner et al., 2013b); such analysis could provide an avenue for future work.

## 5 Conclusion

Monophasic defibrillation shocks in the 5–10 V/cm range may elicit wavefront propagation from the larger blood vessels while the surrounding tissue is relatively refractory; this may either cause post-shock activations (causing failure of the defibrillation shock) or may increase the likelihood of successful defibrillation via wavefront annihilation with the fibrillation waves. Strong monophasic shocks from intracardiac cathodes may elicit low amplitude boundary wave propagation via break excitation from subepicardial vessel VEs, which could suggest an important mechanism of shock failure and reentry re-initiation.

Low-energy defibrillation protocols should focus on eliciting wavefront propagation from vessel VEs surrounded by excitable tissue, as excitation propagation requires low shock strengths [around 1 V/cm, in the regime of low-energy defibrillation (Fenton et al., 2009; Janardhan et al., 2012; Luther et al., 2012; Rantner et al., 2013b)] in this phase of the action potential. The low shock strengths also ensure that no break excitations are elicited from vessel VEs in relatively refractory tissue, as break excitations require shock strengths of greater than 1 V/cm.

Bidomain modeling of defibrillation should include the coronary vasculature, as it is responsible for large magnitude VEs which may affect the fibrillation dynamics. If the vasculature is resolved, the insulating vessel walls should also be resolved as they strongly affect the VEs produced by the vasculature and thus the electrophysiological behavior via the non-linear Hodgkin–Huxley type action potential model.

## Author Contributions

Designed the research: MB and AC. Performed the research: AC. Contributed analytic tools: EV. Wrote the manuscript: AC, MB, and EV.

## Conflict of Interest Statement

The authors declare that the research was conducted in the absence of any commercial or financial relationships that could be construed as a potential conflict of interest.
